# Connexin 43-Based Therapeutics for Dermal Wound Healing

**DOI:** 10.3390/ijms19061778

**Published:** 2018-06-15

**Authors:** Jade Montgomery, Gautam S. Ghatnekar, Christina L. Grek, Kurtis E. Moyer, Robert G. Gourdie

**Affiliations:** 1 Virginia Tech Carilion Research Institute, Roanoke, VA 24016, USA; jmont@vt.edu; 2 School of Biomedical Engineering and Sciences, Virginia Tech-Wake Forest University, Blacksburg, VA 24061, USA; 3 FirstString Research, Inc., Mount Pleasant, SC 29464, USA; ghatnekar@firststringresearch.com (G.S.G.); grek@firststringresearch.com (C.L.G.); 4 Department of Surgery, Virginia Tech Carilion School of Medicine, Roanoke, VA 24016, USA; kemoyer@carilionclinic.org; 5 Department of Surgery, Carilion Clinic, Roanoke, VA 24016, USA; 6 Department of Emergency Medicine, Virginia Tech Carilion School of Medicine, Roanoke, VA 24016, USA

**Keywords:** gap junctions, hemichannels, connexins, skin, wound healing, scar formation, peptide

## Abstract

The most ubiquitous gap junction protein within the body, connexin 43 (Cx43), is a target of interest for modulating the dermal wound healing response. Observational studies found associations between Cx43 at the wound edge and poor healing response, and subsequent studies utilizing local knockdown of Cx43 found improvements in wound closure rate and final scar appearance. Further preclinical work conducted using Cx43-based peptide therapeutics, including alpha connexin carboxyl terminus 1 (αCT1), a peptide mimetic of the Cx43 carboxyl terminus, reported similar improvements in wound healing and scar formation. Clinical trials and further study into the mode of action have since been conducted on αCT1, and Phase III testing for treatment of diabetic foot ulcers is currently underway. Therapeutics targeting connexin activity show promise in beneficially modulating the human body’s natural healing response for improved patient outcomes across a variety of injuries.

## 1. Gap Junctions, Connexins, and Skin Wound Healing

**Figure 1:**
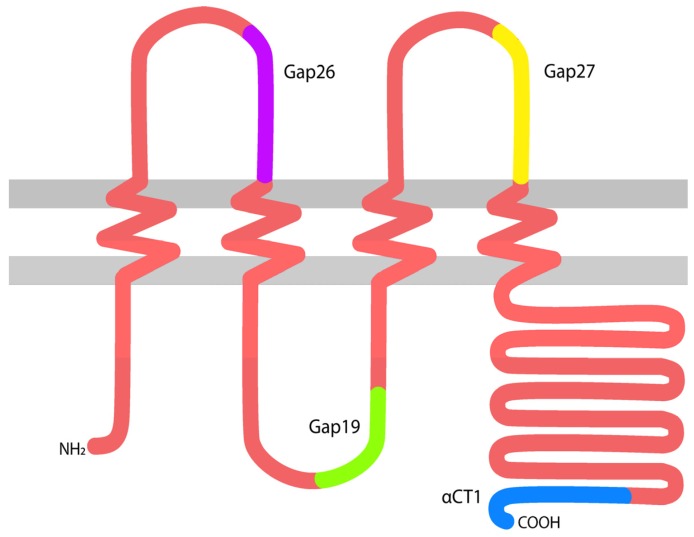
A diagram of connexin 43 (Cx43) spanning the cell membrane, with approximate locations highlighted from which several memetic peptides were derived. αCT1: alpha connexin carboxyl terminus 1.

Gap junctions (GJs) are complexes of intercellular channels composed of proteins encoded by the connexin multigene family [1]. Gap junctions enable direct cytoplasmic coupling between cells, permitting the intercellular exchange of small molecules (<1000 Da). Additionally, undocked gap junctional connexons or hemichannels are also increasingly recognized as having roles in homeostasis and disease [2,3,4,5]. A number of connexin gene family members have been reported to be expressed in skin, including connexin 26 (Cx26), Cx30, Cx31.1, Cx30.3, Cx37, Cx40, Cx45 and Cx43 [6,7,8,9,10]. Of these, Cx43 (Figure 1) is the most abundant and ubiquitous, being present in both the epidermal and dermal cutaneous layers.

Wound healing typically progresses in four stages: Hemostasis, inflammation, proliferation, and maturation [11]. Hemostasis begins immediately after injury, with platelets and various clotting factors invading the wound space to create a fibrin clot that prevents further bleeding. After hemostasis is achieved, inflammation begins within an hour of the original injury. Blood vessels dilate and become more porous, enabling inflammatory leukocytes to invade the wound and phagocytize bacteria and dead/damaged cells. It is this inflammatory stage that is the key target of many wound healing experiments. Fetuses, which do not mount a mature inflammatory response, are able to heal wounds without leaving a lasting scar, particularly during mid-gestational stages [12,13,14]. Research has also found that eliminating the inflammatory response in an adult mouse resulted in wounds that healed efficiently with reduced scarring [15]. On the other hand, failure of the inflammatory phase to resolve appears to be an important aspect of why pathological skin wounds, such as diabetic foot ulcers, are slow to heal [16].

Gap junction channel function and connexin activity have long been recognized as having important assignments in nearly all phases of skin wound healing, including in the coordination of the inflammatory response, propagation of injury signals between cells, wound closure, granulation-tissue formation, and scar remodeling after injury [6,7,8,9,10,17,18,19,20,21,22,23,24,25,26,27,28]. In this short review, we will summarize key findings on basic research into GJs and connexins in skin wound healing as well as recount recent progress on translating this fundamental knowledge to the clinic.

## 2. Early Work on the Role of Cx43 in Cutaneous Wound Healing

A key initial set of findings on the role of connexins in cutaneous wound healing was made in the laboratory of Paul and co-workers [26]. In studies in rodent models, it has been determined that Cx26, Cx31 and Cx43 undergo characteristic cell-specific changes during the wound healing progression [26,27]. Of particular note, Cx43 expression, as well as GJ-mediated intercellular communication, decreases transiently in epidermal cells at the wound edge over the first 24 h following injury [26,27]. Cx43 was downregulated not only in the epidermis of the wound immediately following injury but in the epidermis surrounding the wound as well. A contrasting observation was made in the deep dermis. Here, Cx43 was found to be transiently upregulated in fibroblasts and other tissues in the hours immediately following wounding. After one week, and at later time-points associated with granulation tissue formation and remodeling, increased Cx43 was associated with increases in granulation tissue formation and maturity. Taken together, these results indicated the possibility that localized modulation of Cx43 levels, or certain aspects of Cx43 function, could be a potential method for beneficially altering the cutaneous healing response. This prospect is also suggested by studies of the buccal mucosa, the tissue that lines the inside of the cheeks and floor of the mouth [29], and gingival tissues lining the gum [24,30,31]. In these mucosal tissues, both of which heal more quickly and with significantly less scarring than skin, this relatively fetal-like healing response occurs in association with a rapid and strong downregulation of Cx43.

The first in vivo study of the potential benefits of manipulating wound-localized Cx43 levels was conducted by Green, Becker, and colleagues, who topically applied Cx43 antisense directly to healing wounds [20]. Application of a Cx43 antisense gel to adult rat wounds immediately after wounding increased the rate of Cx43 downregulation in the epidermis and prevented the upregulation of Cx43 in the dermis. This had the remarkable macroscopic effect of reducing inflammation at the wound site, increasing the rate of wound closure, and reducing the appearance of scars at 12 days post-wounding. These effects were particularly noticeable for incisional wounds, although excisional wounds were also improved. The transient upregulation of Cx43 in the smooth muscle cells and endothelial cells of the blood vessels after wounding has been suggested to increase vasodilation and allow the infiltration of inflammatory cells. The Cx43 antisense prevented this upregulation, resulting in a decrease in inflammatory neutrophil numbers and a reduction in the overall inflammatory response in the antisense-treated tissue. The granulation tissue area was also significantly decreased in the treated wounds. In a follow-up study, the group reported decreases in leukocytes and macrophages in Cx43 antisense-treated wounds, concomitant with reduced expression of CC chemokine ligand-2 and Tumor Necrosis Factor alpha, suggesting that the observed enhanced regeneration may have been mediated in part by attenuating inflammation at the wound site [32].

More recently, Martin and co-workers have used an alternate gene knockdown approach, short interfering RNAs (siRNAs) targeted to Cx43, together with the Cx43 mimetic peptide Gap27, to study channel-dependent and independent effects of Cx43 in the response of dermal cells to injury [33]. Their data indicates that the response to targeting Cx43 varies between cell types (keratinocyte versus fibroblast) and between cells of the same type (skin fibroblasts), but of different tissue origins. The knockdown of Cx43 via siRNA enhanced both scrape wound closure and cell proliferation in dermal fibroblasts of human adults and neonates, indicating roles for Cx43 in cell proliferation and migration in these cell types. By contrast, in adult keratinocytes and juvenile foreskin fibroblasts, only scrape wound closure was enhanced, indicating that in these tissue types Cx43 still has a significant effect on cell migration, but its knockdown does not enhance proliferation.

Similar to the Cx43 knockdown/anti-sense experiments, excisional wound studies of a Cx43 heterozygous knockout (Cx43^+/−^) mouse showed decreased inflammation and increased wound closure in the Cx43^+/−^ mouse model compared to wildtype littermates [23]. While no difference was reported in the collagen deposition of the granulation tissue, increased numbers of active dermal fibroblasts were found in the wound space of the Cx43^+/−^ mice, indicating increased fibroblast infiltration/proliferation and activation. Gene expression of extracellular remodeling proteins, including collagen I and III, was also significantly increased in the Cx43^+/−^ mice seven days post-injury; a time-point consistent with the initiation of the remodeling phase of scar tissue. 

Research on chronic wounds, such as venous leg ulcers and diabetic foot ulcers, have shown that Cx43 may be a key participating protein in these conditions [8,17,21,34,35,36,37,38,39,40,41,42]. Studies of diabetic wounds found Cx43 persistence at the wound edge in these chronic wounds [34], while another study by the Becker group on biopsies taken from human venous leg ulcers determined that Cx43 was significantly overexpressed, not just at the edge of these chronic wounds, but throughout the entire dermis of the biopsies [35]. Cx43 over-expression has also been reported to show strong correlations to varicose vein severity in patients—a precursor to leg ulceration from venous insufficiency [36].

## 3. Preclinical Studies of αCT1 Peptide in Skin Wound Healing

During the last decade, a number of peptides targeting specific activities and functions of Cx43 have been developed and studied in the context of wound healing. These include peptides such as alpha connexin carboxyl terminus 1 (αCT1), Gap19, Gap26, Gap27, and more, each targeting different binding sites with varying specificity and size in attempts to narrow down the mode of action and assess therapeutic opportunity [7,8,9,20,30,37,38,39,40,41,42]. Although many of these peptides have been studied in depth for years, only one peptide thus far, αCT1, has moved forward to pivotal Phase III clinical testing (NCT02667327).

αCT1, also referred to as aCT1 or ACT1 in publications, incorporates the last nine amino acids of the Cx43 carboxyl terminus (CT) with an amino terminal antennapedia internalization vector to enable the peptide to penetrate the cell cytoplasm [43]. Originally developed to study the effects of binding between Cx43 and the actin-binding protein zonula occludens-1 (ZO-1), αCT1 peptide competitively inhibits the interaction between the Post synaptic density Drosophila disc large tumor suppressor Zonula occludens-1 (PDZ)-binding domain at the CT of Cx43 and the second PDZ domain of ZO-1 [43,44]. Ongoing work has indicated that the peptide also influences interactions within and/or between Cx43 molecules, with effects on the Cx43 phosphostatus [45,46]. Unlike Cx43 antisense and knockout models, αCT1 does not appear to affect Cx43 protein levels [7,43]. This suggests that its mode of action in wound healing is unlikely to be mediated by the direct effects on the abundance of Cx43, although the influence of αCT1 may well involve downstream alterations in the network of protein-protein interactions that flow from a decrease of Cx43 protein levels at the wound edge. The prevention of Cx43 recycling and transport facilitated by ZO-1 caused by αCT1, as well as non-PDZ based interactions involving αCT1, including binding the Cx43 molecule itself [45], are two likely candidates for causing this alteration.

Early observations in scratch wound assays of 3T3 fibroblasts treated with αCT1 noted that treated fibroblasts appeared more active, migrating with greater speed across the scratch [6]. When mouse excisional wounds were treated with αCT1, inflammation was reduced, and the wound closure rate was increased [7,10,47], mirroring the results of the Cx43 antisense and knockout experiments. Additionally, strength testing analyses of scar tissue 90 days post-wounding revealed that αCT1 treated scars had significantly improved mechanical properties compared to control [7]. Interestingly, the improvement in scar mechanical properties seen at 90 days was more marked than that at 30 days post-wounding, suggesting that the acute treatment by αCT1 had effects that continued long into the remodeling phase of scar formation [7,47]. Additional studies of αCT1 skin wound healing were conducted in a pig model [7]. Porcine models are considered the gold standard for wound healing studies as pig skin is thought to be the closest analogue to human skin [48]. Porcine dermal wounds treated with αCT1 showed decreased granulation tissue area size and increased sub-epidermal vascularity, somewhat regenerating the patterns of blood vessel distribution found in unwounded skin [7].

Additional therapeutic opportunities beyond undiseased dermal wound healing have been identified for αCT1. Topical ophthalmic delivery of αCT1 resulted in decreased inflammation and accelerated healing in both a standard rat model of corneal injury [49] and a diabetic rat model of corneal injury [50]. The Cx43-based peptide has also been found to modulate the biological response to silicone implants, attenuating neutrophil infiltration and increasing vascularity of the specialized internal scar tissue that forms around implants, also reducing the density of activated fibroblasts (myofibroblasts) and type I collagen deposition in this tissue [51]. Application of αCT1 directly to an infarcted heart improved cardiac contractility, reduced the propensity for arrhythmia, and maintained action potential conduction velocity at normal speeds [46,47]. As we will discuss in detail in the following section, treatment of chronic diabetic foot ulcers and venous leg ulcers with αCT1 significantly reduced ulcer size, increased the likelihood of complete ulcer closure, and decreased the time to ulcer closure [37,41].

## 4. Clinical Trials

Given the efficacy of αCT1 in animal studies, multiple Phase I and Phase II clinical trials have since been conducted on the wound healing and scar reduction capabilities of αCT1 [37,38,39,41,42]—see also Table 1. These clinical trials have studied the safety and efficacy of αCT1 in the treatment of venous leg ulcers, diabetic foot ulcers, and surgical wounds, with the peptide being tested as the active ingredient of a gel formulation branded as Granexin^®^.

**Figure 2:**
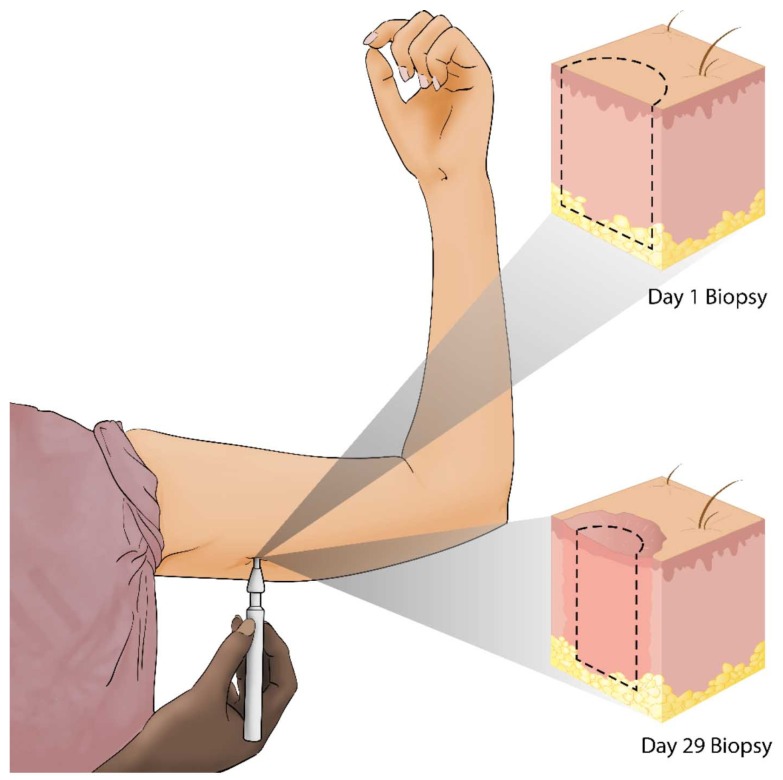
Alpha connexin carboxyl terminus 1 (αCT1) Phase I clinical trial sampling scheme, performed on healthy human volunteers.

The initial clinical trial on the effect of αCT1 on human dermal wound healing was performed on 49 healthy human volunteers in Switzerland in a randomized, double-blind Phase 1 study. As shown in Figure 2, on Day 1 a biopsy punch was used to create a wound in the unblemished skin underneath both arms. One underarm wound from the patient was treated with an αCT1 gel formulation, while the wound on the patient’s other underarm was treated with a vehicle gel, enabling within-patient comparisons. Treatments were applied immediately after injury and again 24 h later. The αCT1 dosage in the gel depended on the cohort. Cohort 1 received a gel with 20 µM of αCT1; Cohort 2, 50 µM; Cohort 3, 100 µM, and Cohort 4, 200 µM of αCT1. Cohort 3, which received a gel with 100 µM of αCT1, was given the same dosage that was used in Phase II clinical trials and is considered the therapeutic dosage. Wound healing was followed for 29 days and recorded photographically. On Day 29, following final photograph collection, the healed scars were biopsied to permit examination of the histological features of the scar tissue. The biopsies were washed, placed in paraformaldehyde for 24 h, and embedded in paraffin for sectioning. Data collected from these biopsy sections revealed improved healing outcomes, in terms of collagen order, density and maturity, with αCT1 treatment and has formed the basis of ongoing studies into the αCT1 mode of action. Importantly, from the perspective of the safety focus of Phase I clinical testing, αCT1 usage showed no local or systemic adverse effects associated with treatment.

Continuing onto Phase II, two of the Phase II clinical trials involved studies of chronic skin wounds characterized by chronic inflammation and retarded re-epithelialization: Venous leg ulcers and diabetic foot wounds [37,41]. For treatment of chronic wounds, αCT1 was topically applied to the wound area twice during the first week, and then on a weekly basis thereafter. In the venous leg ulcer trial, *n* = 92 patients were randomized for study [37]. αCT1 treatment was associated with a significantly greater reduction in the mean percent ulcer area by 12 weeks (79% wound closure in the treatment group compared to 36% in the control), and a doubling in incidence of complete wound closure by 12 weeks (57% of the treatment group had completely healed wounds by the study end point of 12 weeks compared to only 28% of the control group). Venous leg wounds treated with αCT1 also showed a shorter time to 50% (*p* = 0.014), and 100% (*p* = 0.041) wound closure than the control group. The median time to 50% and 100% wound closure in the treatment group was 2.9 and 6.0 weeks, respectively, while the control group took an average of 6.9 and 12.1 weeks to reach the same milestones.

Similar results were found for diabetic foot ulcers in a separate clinical trial, in which *n* = 92 patients were randomized for study [41]. The mean percent ulcer area at 12 weeks was 94% for the treatment group versus 52% for the control, and the incidence of 100% wound closure at the study end point of 12 weeks was 81% for treatment versus 50% for control. Median time to complete wound closure was 6.0 weeks for the treatment group while the control group’s estimated median time to closure was 14.6 weeks. Time to 50% ulcer closure for this study was not significantly different. To summarize the results of these two clinical trials, chronic wounds treated with αCT1 heal more quickly and are more likely to completely resolve within 12 weeks than the control group of chronic wounds treated with the current standard of care only.

**Figure 3:**
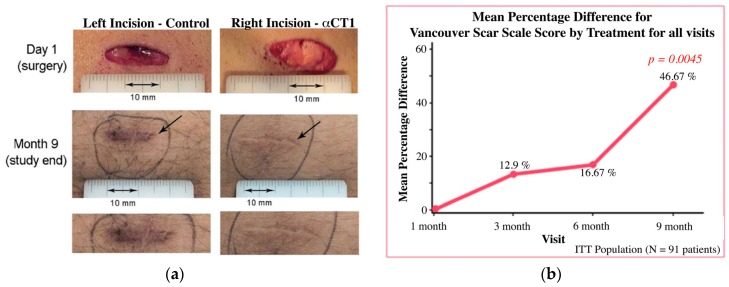
(**a**) Photographs of a single patient’s αCT1 and control treated wounds immediately after surgery and at the study end point of nine months; (**b**) Mean percentage difference between treatment and control scar scores for the Phase II scar appearance clinical trials [35].

In a third Phase II clinical trial, the potential of αCT1 in reducing post-surgical scarring was assessed [39]. Unlike the weekly αCT1 application protocol applied in the treatment of chronic wounds, αCT1 was applied to surgical wounds immediately after injury and then again 24 h later. This treatment regime was followed by scarring assessment over a nine-month study period. This acute treatment regime was similar to that used in the earlier animal studies involving therapeutic evaluation of the Cx43 antisense as well as αCT1 [7,20]. The clinical trial involved 91 patients who had received laparoscopic abdominal surgery involving two or more incisions, allowing within-patient controls with the surgical wound on one side of the abdomen treated with αCT1 and the control wound on the opposite side. Treatment versus control was randomized to patient sides, and both wounds were treated with identical conventional standard of care protocols. [39]. Scar appearance, as judged by the Vancouver Scar Scale clinical standard, was equivalent between treatment groups after one month, but after nine months αCT1 treated scars showed a highly significant, 47%, improvement (*p* < 0.005) in scar appearance (Figure 3). Since αCT1 was only applied in the first two days, the results raise interesting questions about the mechanism by which brief, transitory targeting of Cx43 and/or its activities after injury is able to induce a long-term modification in scarring outcome. 

**Figure 4:**
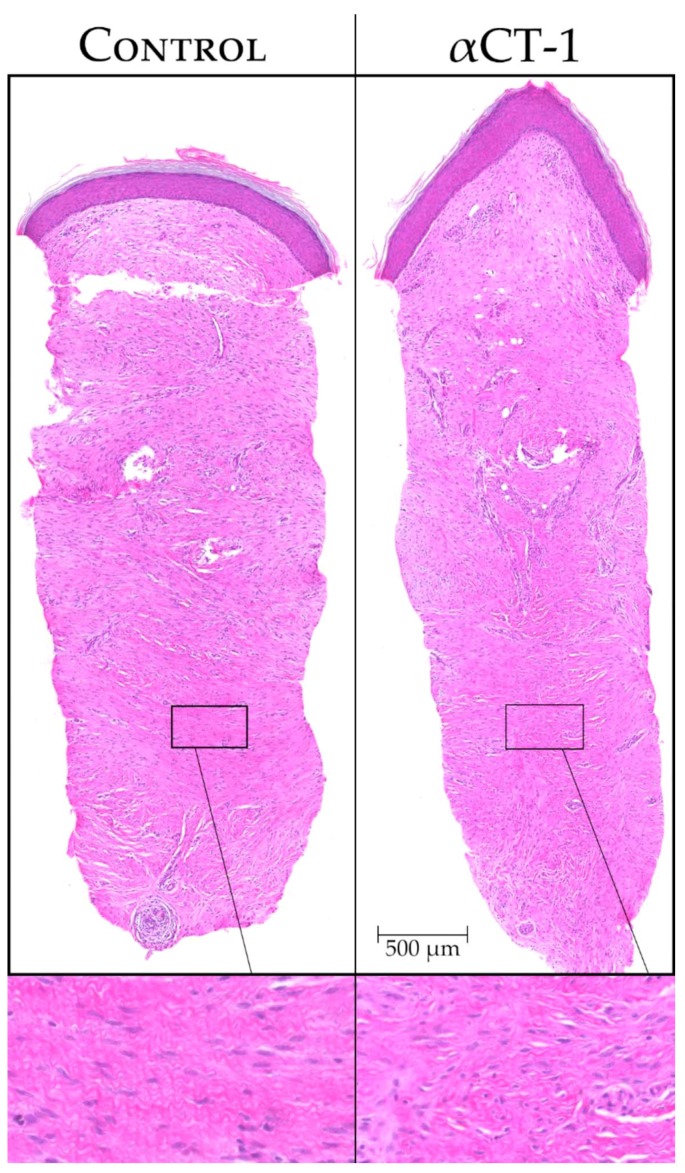
H&E stained whole sections of Phase I biopsies from a single patient at 29 days post-wounding. The left arm was treated with a vehicle (**left**), while the right arm was treated with 100 μM αCT1 (**right**). The boxed regions highlight areas of subtle variance in tissue organization deep within the dermis between the control and treated scars—magnified 4.67× from upper panels.

Preclinical results found improvements in the tensile strength of αCT1 treated wounds—a property directly linked to extracellular matrix composition and structure [7,47]. In line with this, we have identified structural changes in the extracellular matrix of αCT1 treated wounds from biopsies collected in Phase I clinical trial that suggest the peptide is prompting the deposition of an initial collagen matrix more similar to unwounded skin (Figure 4). Work exploring this hypothesis is currently ongoing.

## 5. Conclusions

Preclinical and clinical studies of αCT1 have indicated that this peptide, based on the CT-most sequence of Cx43, beneficially modulates the healing of both undiseased and diseased, chronic skin wounds without detrimental side-effects. However, important work remains to be undertaken to characterize the details of the molecular and cellular mechanisms by which therapies targeting Cx43 function, such as αCT1, improve wound healing and mitigate scar formation. Identifying key parts of the cascade may allow the development of improved, targeted therapeutics, reducing the potential for off-target effects. Connexin-based therapeutics are also being explored in preclinical studies of injury to other organ systems and tissues, including the heart, eye, brain, and lungs [52,53,54,55,56,57,58,59]. Moving from topical delivery of drugs like αCT1 to internal administration in tissues such as the heart or brain will pose significant challenges. The regulatory bar for safety will be necessarily higher. There will also be questions on optimal route and mode of delivery, treatment regime, the stability of the drug in body fluids, the negotiation of the immune system, and obstacles such as the blood-brain barrier that will need to be addressed. This being said, ongoing research provides encouragement that connexin-based therapeutics could be a promising path towards future medical interventions in the healing of the human body.

## Figures and Tables

**Table 1:** Summary of completed alpha connexin carboxyl terminus 1 (αCT1) clinical trials.

Clinical Trial Phase	Phase I	Phase II		
Wound Type	Healthy Human Dermal Wounds	Venous Leg Ulcers	Diabetic Foot Ulcers	Cutaneous Scarring/Laparoscopic Incisions
Treatment Regimen	Immediately after wounding and 24 h later	Twice during the 1st week and once a week thereafter	Twice during the 1st week and once a week thereafter	Immediately after wounding and 24 h later
Patients	49	92	92	91
No Adverse Effects	✓	✓	✓	✓
Mean Percent Ulcer Area Reduction at 12 Weeks	-	79% αCT1 vs. 36% control	94% αCT1 vs. 52% control	-
Incidence of Complete Ulcer Closure at 12 Weeks	-	57% αCT1 vs. 28% control	81% αCT1 vs. 50% control	-
Comparative Vancouver Scar Scale Scores at 9 Months	-	-	-	47% better for αCT1 compared to within-patient controls

## Abbreviations

Cx43	Connexin 43
αCT1	Alpha connexin carboxyl terminus 1
GJ	Gap Junction
ZO-1	Zonula occludens-1
CT	Carboxyl terminus
